# Supplementary material for: Impact of delayed sternal closure on wound infections following neonatal and infant cardiac surgery
Source: PLoS One. 2022 May 23;17(5):e0267985. doi: 10.1371/journal.pone.0267985 (PMC9126390; doi:10.1371/journal.pone.0267985)
Supplement: S1 Dataset — key—patient number for anonymization; DSC—delayed sternal closure (1—delayed sternal closure, 0—primary sternal closure); TUSC—time until DSC (days), Gender (0—female; 1—male); OR_age (age at index operation in days), ECMO—extracorporeal membrane oxygenation (1—ECMO; 0—no ECMO); CPB—cardiopulmonary bypass (1—CPB; 0—no CPB); Re_Ex (Chest Re-Exploration, 1—yes, 0—no, 99—no data available); ReOP—Redo (1—Reoperation, 0—no reoperation), STS-EACTS—STAT Mortality category, Mortality categories for congenital heart surgery from the Society of Thoracic Surgeons-European Association for Cardio-Thoracic Surgery; Aristotle—Aristotle Score—Aristotle Score (Simple–to very complex. 1–15); surg_mort—mortality (1—yes, 0—no). (PDF) [file pone.0267985.s001.pdf]

Supporting information 1. Data set of all 358 patients enclosed in this study.

key	DSC	TUSC	Gender	SWI	OR_age	ECMO	CBP	Re_Ex	ReOP	STS_EACTS	Aristotle	surg_mort
207	1	11	0	0	131	1	1	0	1	4	9,50	1
249	1	4	1	1	9	0	1	0	0	4	7,00	0
355	1	19	0	0	1	1	0	0	0	4	6,00	1
330	1	3	1	0	9	0	1	0	0	5	15,00	0
334	1	27	0	0	179	1	1	0	1	5	14,50	1
340	1	14	0	0	44	1	1	1	0	5	14,50	1
342	1	8	0	1	29	1	1	0	0	5	14,50	0
346	1	7	1	0	16	1	1	0	0	5	14,50	0
348	1	2	0	0	14	0	1	0	0	5	14,50	0
335	1	3	1	0	13	1	1	0	0	5	14,50	0
339	1	28	1	0	11	1	1	0	0	5	14,50	0
345	1	7	0	0	11	1	1	0	0	5	14,50	1
341	1	12	1	0	8	1	1	0	0	5	14,50	0
338	1	25	0	0	6	1	1	0	0	5	14,50	0
347	1	9	1	0	5	1	1	0	0	5	14,50	1
336	1	5	1	0	4	1	1	0	0	5	14,50	0
337	1	31	1	0	4	1	1	0	0	5	14,50	0
343	1	6	1	0	3	1	1	0	0	5	14,50	1
213	1	3	1	0	149	0	1	0	1	5	9,50	0
344	1	8	0	1	13	1	1	0	0	5	9,50	0
128	1	5	1	0	17	0	1	0	0	4	13,00	0
127	1	5	0	1	8	1	1	0	0	4	13,00	0
129	1	5	1	0	3	0	1	0	0	4	13,00	0
351	1	1	0	0	120	0	1	0	0	4	12,50	0
350	1	3	1	0	68	0	1	0	0	4	12,50	0

214	1	2	0	0	276	0	1	0	0	4	11,00	0
221	1	1	0	0	221	0	1	0	0	4	11,00	0
333	1	2	1	0	164	0	1	0	0	4	11,00	0
218	1	8	1	0	161	0	1	0	0	4	11,00	1
327	1	9	1	0	93	1	1	0	0	4	11,00	1
331	1	6	1	0	63	0	1	0	0	4	11,00	0
210	1	12	1	0	59	1	1	0	0	4	11,00	0
125	1	5	0	0	27	1	1	0	0	4	11,00	1
126	1	4	1	0	14	1	1	0	0	4	11,00	0
131	1	4	1	0	14	0	1	0	0	4	11,00	0
124	1	6	1	0	13	1	1	0	0	4	11,00	0
332	1	2	0	1	11	0	1	0	0	4	11,00	0
326	1	7	1	0	11	1	1	0	0	4	11,00	1
328	1	8	0	0	10	1	1	0	0	4	11,00	1
122	1	13	1	0	9	1	1	0	0	4	11,00	0
123	1	12	1	0	7	1	1	0	0	4	11,00	0
130	1	1	1	0	5	0	1	0	1	4	11,00	0
318	1	3	1	0	1	0	1	0	1	4	11,00	0
329	1	5	0	0	1	1	1	0	0	4	11,00	0
309	1	1	0	0	19	0	1	0	0	4	10,80	0
215	1	3	1	0	93	0	1	0	1	4	10,00	1
280	1	2	1	0	91	0	1	0	0	4	10,00	0
277	1	1	0	0	63	0	1	0	1	4	10,00	0
281	1	2	1	0	42	0	1	0	0	4	10,00	0
275	1	2	1	0	26	0	1	0	0	4	10,00	0
274	1	2	1	0	18	0	1	0	0	4	10,00	0
282	1	2	1	0	17	0	1	0	0	4	10,00	0
283	1	2	1	0	16	0	1	0	0	4	10,00	0

270	1	1	1	0	16	0	1	0	0	4	10,00	0
266	1	5	1	0	14	1	1	0	0	4	10,00	1
285	1	4	1	0	13	0	1	0	0	4	10,00	0
268	1	3	1	0	12	1	1	0	0	4	10,00	1
267	1	5	1	1	12	1	1	0	0	4	10,00	0
288	1	2	1	0	11	0	1	0	0	4	10,00	0
273	1	5	0	1	9	0	1	0	0	4	10,00	0
286	1	4	0	1	9	0	1	0	0	4	10,00	0
271	1	2	1	0	9	0	1	0	0	4	10,00	0
276	1	3	1	0	9	0	1	0	0	4	10,00	0
278	1	4	1	0	9	0	1	0	0	4	10,00	0
211	1	9	0	1	9	1	1	0	0	4	10,00	0
113	1	6	1	0	8	0	1	0	0	4	10,00	0
272	1	4	1	0	7	0	1	0	0	4	10,00	0
279	1	2	0	0	7	0	1	0	0	4	10,00	0
120	1	7	0	0	7	0	1	1	0	4	10,00	0
269	1	7	0	0	7	0	1	0	0	4	10,00	0
284	1	3	1	1	6	0	1	0	0	4	10,00	0
265	1	25	1	1	4	1	1	0	0	4	10,00	0
287	1	5	1	0	4	0	1	0	0	4	10,00	0
208	1	15	1	0	180	1	1	0	1	4	9,50	1
216	1	1	1	1	163	0	1	0	0	4	9,00	0
321	1	3	0	0	118	0	1	0	1	4	9,00	0
317	1	4	0	0	43	0	1	0	0	4	9,00	0
313	1	12	0	1	29	1	1	1	0	4	9,00	1
315	1	4	1	0	21	0	1	0	0	4	9,00	0
311	1	10	1	0	17	1	1	0	0	4	9,00	1
319	1	3	1	0	11	0	1	0	0	4	9,00	0

316	1	1	0	0	7	0	1	0	0	4	9,00	0
320	1	2	0	1	7	0	1	0	0	4	9,00	0
312	1	14	1	0	5	1	1	0	0	4	9,00	0
314	1	5	1	0	2	0	1	0	0	4	9,00	0
310	1	1	1	0	1	1	1	0	0	4	9,00	1
356	1	6	0	0	14	0	1	0	0	4	8,00	0
217	1	1	0	0	230	0	1	0	0	4	7,00	0
247	1	9	0	0	199	1	0	1	1	4	7,00	1
250	1	2	0	1	51	0	0	0	0	4	7,00	0
140	1	2	1	0	202	0	1	1	1	4	6,80	0
154	1	1	1	0	158	0	0	0	0	4	6,80	0
146	1	3	1	0	77	0	1	0	0	4	6,80	0
152	1	1	0	0	72	0	0	0	0	4	6,80	0
149	1	2	1	0	40	0	1	0	0	4	6,80	0
141	1	2	1	0	36	0	1	0	0	4	6,80	0
150	1	3	0	0	19	0	1	0	0	4	6,80	0
138	1	6	1	0	16	0	1	0	0	4	6,80	0
151	1	3	1	0	16	0	1	0	0	4	6,80	0
153	1	2	0	0	14	0	0	0	0	4	6,80	0
155	1	1	0	0	13	0	1	0	0	4	6,80	0
133	1	6	1	0	12	1	1	0	0	4	6,80	1
134	1	12	0	0	12	1	1	0	0	4	6,80	0
142	1	1	0	0	11	0	1	0	0	4	6,80	0
147	1	4	1	0	10	0	1	0	0	4	6,80	0
143	1	2	1	1	9	0	1	0	0	4	6,80	0
145	1	3	1	1	9	0	1	0	0	4	6,80	0
139	1	5	1	0	9	0	1	0	0	4	6,80	1
144	1	9	0	0	8	0	1	0	0	4	6,80	0

136	1	8	0	0	6	1	1	0	0	4	6,80	0
148	1	4	1	1	5	0	1	0	0	4	6,80	0
135	1	32	1	0	4	1	0	0	0	4	6,80	0
137	1	9	1	0	3	1	0	0	0	4	6,80	1
352	1	24	0	0	112	1	0	0	0	4	6,00	1
189	1	5	1	0	63	0	0	0	0	4	6,00	0
190	1	1	1	0	49	0	0	0	0	4	6,00	0
188	1	1	1	0	17	0	0	0	0	4	6,00	0
353	1	7	1	0	16	1	0	0	0	4	6,00	0
198	1	2	1	0	14	0	1	0	0	4	6,00	0
201	1	5	0	0	13	0	0	0	0	4	6,00	0
200	1	4	0	0	9	0	0	0	0	4	6,00	1
354	1	12	0	0	8	1	0	0	0	4	6,00	0
197	1	11	1	0	7	0	1	0	0	4	6,00	1
199	1	1	1	1	6	0	0	0	0	4	6,00	0
349	1	2	1	0	189	0	1	0	1	3	11,00	0
109	1	5	0	0	37	0	1	0	0	3	10,00	0
117	1	2	0	0	27	0	1	0	0	3	10,00	0
107	1	1	1	1	18	0	1	0	0	3	10,00	0
115	1	1	1	0	17	0	1	0	0	3	10,00	0
101	1	4	1	0	16	1	1	0	0	3	10,00	0
114	1	1	1	1	15	0	1	0	0	3	10,00	0
108	1	2	1	0	14	0	1	0	0	3	10,00	0
104	1	1	1	0	13	0	1	0	0	3	10,00	0
116	1	2	1	0	13	0	1	0	0	3	10,00	0
111	1	5	0	1	11	0	1	0	0	3	10,00	0
102	1	1	1	0	11	0	1	0	0	3	10,00	0
119	1	3	1	0	8	0	1	0	0	3	10,00	0

105	1	5	1	0	7	0	1	0	0	3	10,00	0
110	1	3	1	0	6	0	1	0	0	3	10,00	0
112	1	1	1	0	6	0	1	0	0	3	10,00	0
106	1	1	1	1	4	0	1	0	0	3	10,00	0
118	1	2	0	0	4	0	1	0	0	3	10,00	0
103	1	2	1	0	3	0	1	0	0	3	10,00	0
163	1	7	1	0	258	1	1	1	0	3	9,00	1
161	1	7	0	0	214	1	1	0	1	3	9,00	1
166	1	3	1	0	180	0	1	0	0	3	9,00	0
170	1	3	0	0	167	0	1	0	0	3	9,00	0
169	1	1	1	0	156	0	1	0	0	3	9,00	0
164	1	2	0	0	136	0	1	1	1	3	9,00	0
167	1	3	0	0	129	0	1	0	0	3	9,00	0
168	1	2	1	1	107	0	1	0	0	3	9,00	0
165	1	5	0	0	64	0	1	0	0	3	9,00	0
162	1	16	1	0	13	1	1	0	0	3	9,00	1
74	1	2	0	0	297	0	1	1	1	2	8,00	0
71	1	13	1	0	171	0	1	0	0	2	8,00	0
72	1	3	0	0	153	0	1	0	1	2	8,00	0
73	1	1	1	0	132	0	1	0	0	2	8,00	0
219	1	1	1	0	82	0	1	0	0	2	8,00	0
220	1	3	0	0	33	0	1	0	0	2	8,00	0
212	1	1	0	0	160	1	1	0	0	1	8,00	1
209	1	15	1	0	5	1	1	0	0	1	8,00	0
1	1	1	1	0	232	0	1	0	1	1	6,00	0
3	1	1	1	0	109	0	1	0	0	1	6,00	0
289	0	99	0	0	99	0	1	0	0	4	11,00	0
296	0	99	1	0	304	0	1	99	0	4	10,00	0

227	0	99	0	0	209	0	1	99	1	4	10,00	0
237	0	99	0	0	165	0	1	99	1	4	10,00	0
291	0	99	1	0	136	0	1	99	1	4	10,00	0
295	0	99	0	0	135	0	1	99	0	4	10,00	0
290	0	99	0	0	72	0	1	99	0	4	10,00	0
294	0	99	0	0	53	0	1	99	0	4	10,00	0
293	0	99	0	0	30	0	1	99	0	4	10,00	0
292	0	99	0	0	4	0	1	99	1	4	10,00	0
323	0	99	1	0	21	0	1	99	0	4	9,00	0
324	0	99	1	0	8	0	1	99	0	4	9,00	0
357	0	99	0	0	333	0	1	99	1	4	8,00	0
322	0	99	1	0	247	0	1	99	1	4	8,00	0
264	0	99	0	0	316	0	1	99	1	4	7,00	0
261	0	99	0	0	314	0	1	99	0	4	7,00	0
262	0	99	0	0	240	0	1	99	1	4	7,00	0
254	0	99	0	0	229	0	1	99	1	4	7,00	0
259	0	99	1	0	223	0	1	99	1	4	7,00	0
260	0	99	1	0	207	0	1	99	1	4	7,00	0
253	0	99	1	0	198	0	1	99	1	4	7,00	0
248	0	99	0	0	192	1	1	99	1	4	7,00	0
263	0	99	1	0	167	0	1	99	1	4	7,00	0
256	0	99	0	0	152	0	1	99	1	4	7,00	0
252	0	99	1	0	147	0	1	99	1	4	7,00	0
255	0	99	1	0	139	0	1	99	1	4	7,00	0
257	0	99	1	0	137	0	1	99	1	4	7,00	0
251	0	99	1	1	134	0	1	99	1	4	7,00	0
258	0	99	1	0	112	0	1	99	1	4	7,00	0
157	0	99	1	0	44	0	1	99	0	4	6,80	0

132	0	99	1	0	27	0	1	99	1	4	6,80	0
158	0	99	1	0	20	0	0	99	0	4	6,80	0
160	0	99	0	0	15	0	1	99	0	4	6,80	0
159	0	99	0	0	12	0	0	99	0	4	6,80	0
156	0	99	1	0	9	0	1	99	0	4	6,80	0
192	0	99	0	0	363	0	1	99	1	4	6,00	0
243	0	99	0	0	299	0	0	99	0	4	6,00	0
245	0	99	1	0	153	0	1	99	1	4	6,00	0
240	0	99	1	0	140	0	1	99	1	4	6,00	0
244	0	99	0	0	83	0	1	99	1	4	6,00	0
194	0	99	0	0	42	0	0	99	0	4	6,00	0
196	0	99	1	0	37	0	0	99	0	4	6,00	0
193	0	99	0	0	32	0	0	99	0	4	6,00	0
195	0	99	1	0	18	0	0	99	0	4	6,00	0
206	0	99	0	0	18	0	0	99	0	4	6,00	0
191	0	99	1	0	14	0	0	99	0	4	6,00	0
204	0	99	1	0	11	0	0	99	0	4	6,00	0
202	0	99	1	0	6	0	0	99	0	4	6,00	0
205	0	99	0	0	5	0	0	99	0	4	6,00	0
203	0	99	0	0	4	0	0	99	0	4	6,00	0
238	0	99	1	0	79	0	0	99	1	3	11,00	0
121	0	99	0	0	9	0	1	99	0	3	10,00	0
176	0	99	0	0	355	0	1	99	0	3	9,00	0
171	0	99	0	0	307	0	1	99	1	3	9,00	0
181	0	99	1	0	275	0	1	99	0	3	9,00	0
172	0	99	1	0	234	0	1	99	1	3	9,00	0
178	0	99	0	0	215	0	1	99	0	3	9,00	0
186	0	99	1	0	211	0	1	99	0	3	9,00	0

174	0	99	1	0	199	0	1	99	0	3	9,00	0
184	0	99	0	0	179	0	1	99	0	3	9,00	0
177	0	99	1	0	178	0	1	99	0	3	9,00	0
183	0	99	0	0	177	0	1	99	0	3	9,00	0
179	0	99	1	0	176	0	1	99	0	3	9,00	0
185	0	99	0	0	174	0	1	99	0	3	9,00	0
173	0	99	1	0	164	0	1	99	0	3	9,00	0
175	0	99	0	0	162	0	1	99	0	3	9,00	0
180	0	99	1	0	136	0	1	99	0	3	9,00	0
182	0	99	1	0	106	0	1	99	0	3	9,00	0
239	0	99	1	0	284	0	1	99	1	2	11,00	0
230	0	99	0	0	79	0	1	99	0	2	11,00	0
229	0	99	1	0	167	0	1	99	0	2	10,00	0
241	0	99	1	0	91	0	1	99	0	2	9,00	0
233	0	99	0	0	325	0	1	99	1	2	8,80	0
236	0	99	1	0	239	0	1	99	0	2	8,80	0
231	0	99	1	0	148	0	1	99	1	2	8,80	0
232	0	99	0	0	62	0	1	99	1	2	8,80	0
234	0	99	0	0	8	0	1	99	0	2	8,80	0
94	0	99	1	0	289	0	1	99	0	2	8,00	0
81	0	99	0	0	279	0	1	99	1	2	8,00	0
88	0	99	0	1	277	0	1	99	1	2	8,00	0
80	0	99	1	0	254	0	1	99	0	2	8,00	0
82	0	99	1	0	233	0	1	99	1	2	8,00	0
98	0	99	0	0	220	0	1	99	0	2	8,00	0
97	0	99	1	0	206	0	1	99	0	2	8,00	0
99	0	99	0	0	205	0	1	99	0	2	8,00	0
85	0	99	0	0	202	0	1	99	1	2	8,00	0

90	0	99	1	0	192	0	1	99	0	2	8,00	0
89	0	99	1	0	180	0	1	99	0	2	8,00	0
77	0	99	0	0	171	0	1	99	0	2	8,00	0
84	0	99	0	0	170	0	1	99	1	2	8,00	0
228	0	99	0	0	167	0	1	99	0	2	8,00	0
92	0	99	1	0	163	0	1	99	0	2	8,00	0
96	0	99	0	0	161	0	1	99	0	2	8,00	0
86	0	99	1	0	148	0	1	99	1	2	8,00	0
93	0	99	0	0	146	0	1	99	0	2	8,00	0
83	0	99	0	0	145	0	1	99	1	2	8,00	0
76	0	99	1	0	127	0	1	99	1	2	8,00	0
100	0	99	1	0	124	0	1	99	0	2	8,00	0
79	0	99	1	0	116	0	1	99	0	2	8,00	0
95	0	99	1	0	116	0	1	99	0	2	8,00	0
87	0	99	1	0	113	0	1	99	1	2	8,00	0
91	0	99	0	0	89	0	1	99	0	2	8,00	0
75	0	99	0	0	83	0	1	99	0	2	8,00	0
78	0	99	0	0	40	0	1	99	0	2	8,00	0
5	0	99	1	0	352	0	1	99	1	2	6,00	0
6	0	99	1	0	235	0	1	99	1	2	6,00	0
8	0	99	1	0	151	0	1	99	0	2	6,00	0
246	0	99	1	0	40	0	0	99	0	2	4,00	0
298	0	99	0	0	188	0	1	99	0	2	3,80	0
225	0	99	0	0	33	0	0	99	0	2	3,00	0
223	0	99	0	0	19	0	0	99	0	2	3,00	0
226	0	99	1	0	4	0	0	99	0	2	3,00	0
235	0	99	0	0	223	0	1	99	1	1	10,00	0
187	0	99	1	0	109	0	1	99	0	1	9,00	0

325	0	99	0	0	11	0	1	99	0	1	9,00	0
27	0	99	1	0	365	0	1	99	0	1	6,00	0
15	0	99	1	0	361	0	1	99	0	1	6,00	0
11	0	99	1	0	346	0	1	99	0	1	6,00	0
39	0	99	1	0	344	0	1	99	0	1	6,00	0
61	0	99	1	0	313	0	1	99	0	1	6,00	0
60	0	99	1	0	309	0	1	99	0	1	6,00	0
10	0	99	0	0	305	0	1	99	0	1	6,00	0
23	0	99	1	0	301	0	1	99	0	1	6,00	0
68	0	99	0	0	277	0	1	99	0	1	6,00	0
70	0	99	1	0	270	0	1	99	0	1	6,00	0
29	0	99	0	0	261	0	1	99	0	1	6,00	0
69	0	99	0	0	260	0	1	99	0	1	6,00	0
36	0	99	0	0	256	0	1	99	0	1	6,00	0
40	0	99	0	0	248	0	1	99	0	1	6,00	0
20	0	99	1	0	246	0	1	99	0	1	6,00	0
58	0	99	1	0	246	0	1	99	0	1	6,00	0
34	0	99	1	0	238	0	1	99	0	1	6,00	0
55	0	99	1	0	234	0	1	99	0	1	6,00	0
47	0	99	1	0	233	0	1	99	0	1	6,00	0
66	0	99	0	0	233	0	1	99	0	1	6,00	0
64	0	99	0	0	228	0	1	99	1	1	6,00	0
32	0	99	0	0	217	0	1	99	0	1	6,00	0
42	0	99	0	0	211	0	1	99	0	1	6,00	0
7	0	99	1	0	209	0	1	99	1	1	6,00	0
18	0	99	1	0	203	0	1	99	0	1	6,00	0
13	0	99	0	0	198	0	1	99	0	1	6,00	0
43	0	99	0	0	198	0	1	99	0	1	6,00	0

53	0	99	0	0	197	0	1	99	0	1	6,00	0
63	0	99	0	0	197	0	1	99	0	1	6,00	0
54	0	99	1	0	189	0	1	99	0	1	6,00	0
46	0	99	0	0	183	0	1	99	0	1	6,00	0
26	0	99	1	0	179	0	1	99	0	1	6,00	0
21	0	99	1	0	177	0	1	99	0	1	6,00	0
45	0	99	0	0	176	0	1	99	0	1	6,00	0
41	0	99	1	0	175	0	1	99	0	1	6,00	0
62	0	99	1	0	174	0	1	99	0	1	6,00	0
65	0	99	1	0	172	0	1	99	1	1	6,00	0
2	0	99	1	0	168	0	1	99	0	1	6,00	0
37	0	99	0	0	166	0	1	99	0	1	6,00	0
25	0	99	1	0	165	0	1	99	0	1	6,00	0
48	0	99	0	0	165	0	1	99	0	1	6,00	0
49	0	99	1	0	156	0	1	99	0	1	6,00	0
9	0	99	1	0	155	0	1	99	0	1	6,00	0
67	0	99	1	0	150	0	1	99	0	1	6,00	0
52	0	99	1	0	148	0	1	99	0	1	6,00	0
30	0	99	0	0	142	0	1	99	0	1	6,00	0
50	0	99	0	0	142	0	1	99	0	1	6,00	0
56	0	99	1	0	142	0	1	99	0	1	6,00	0
14	0	99	1	0	139	0	1	99	0	1	6,00	0
24	0	99	1	0	138	0	1	99	0	1	6,00	0
19	0	99	1	0	137	0	1	99	0	1	6,00	0
4	0	99	0	0	135	0	1	99	0	1	6,00	0
12	0	99	0	0	135	0	1	99	0	1	6,00	0
17	0	99	1	0	132	0	1	99	0	1	6,00	0
59	0	99	0	0	130	0	1	99	0	1	6,00	0

22	0	99	1	0	119	0	1	99	0	1	6,00	0
28	0	99	0	0	118	0	1	99	0	1	6,00	0
51	0	99	0	0	108	0	1	99	0	1	6,00	0
16	0	99	1	0	100	0	1	99	0	1	6,00	0
57	0	99	1	0	98	0	1	99	0	1	6,00	0
44	0	99	0	0	95	0	1	99	0	1	6,00	0
35	0	99	0	0	94	0	1	99	0	1	6,00	0
33	0	99	1	0	83	0	1	99	0	1	6,00	0
38	0	99	1	0	69	0	1	99	0	1	6,00	0
31	0	99	1	0	66	0	1	99	0	1	6,00	0
242	0	99	1	0	38	0	1	99	0	1	6,00	0
358	0	99	0	0	149	0	1	99	0	1	5,00	0
299	0	99	0	0	315	0	1	99	0	1	3,00	0
306	0	99	1	0	273	0	1	99	0	1	3,00	0
304	0	99	1	0	266	0	1	99	0	1	3,00	0
297	0	99	1	0	262	0	1	99	0	1	3,00	0
305	0	99	0	0	207	0	1	99	0	1	3,00	0
307	0	99	0	0	189	0	1	99	0	1	3,00	0
303	0	99	1	0	182	0	1	99	0	1	3,00	0
301	0	99	1	0	179	0	1	99	0	1	3,00	0
224	0	99	1	0	154	0	0	99	1	1	3,00	0
308	0	99	0	0	129	0	1	99	0	1	3,00	0
300	0	99	1	0	116	0	1	99	0	1	3,00	0
302	0	99	0	0	111	0	1	99	0	1	3,00	0
222	0	99	0	0	70	0	0	99	1	1	3,00	0

Caption:

key - patient number for anonymization;

DSC - delayed sternal closure (1 - delayed sternal closure, 0 - primary sternal closure);
TUSC - time until DSC (days), Gender (0 - female; 1 - male);
OR_age (age at index operation in days),
ECMO - extracorporeal membrane oxygenation (1 - ECMO; 0 - no ECMO);
CPB - cardiopulmonary bypass (1 - CPB; 0 - no CPB);
Re_Ex (Chest Re-Exploration, 1 - yes, 0 - no, 99 - no data available);
ReOP - Redo (1 - Reoperation, 0 - no reoperation),
STS-EACTS - STAT Mortality category,
Mortality categories for congenital heart surgery from the Society of Thoracic Surgeons-European Association for Cardio-Thoracic Surgery;
Aristotle - Aristotle Score - Aristotle Score (Simple – to very complex. 1 – 15); surg_mort - mortality (1 - yes, 0 - no)
