# Supplementary material for: Impact of delayed sternal closure on wound infections following neonatal and infant cardiac surgery
Source: PLoS One. 2022 May 23;17(5):e0267985. doi: 10.1371/journal.pone.0267985 (PMC9126390; doi:10.1371/journal.pone.0267985)
Supplement: Attachment — Submitted filename: PLOS_Response_to_Reviewer.docx [file pone.0267985.s002.docx]

**Dr. med. Daniel Biermann**

Department of Congenital and Pediatric Heart Surgery

Children‘s Heart Clinic

University Medical Center Hamburg-Eppendorf

Martinistrasse 52

20251 Hamburg

Office: +49(0)40 7410 58221

Mail: d.biermann@uke.de

www.uke.de

Hamburg, 08.03.2022

**Response letter to the Reviewer**

Dear PLOS ONE-editors,

Thank you very much for giving us the opportunity to re-submit our recent research article entitled „Impact of delayed sternal closure on wound infections following neonatal and infant cardiac surgery”.

We very much appreciate the critical review and thoughtful comments on our manuscript and are happy to hear that it is of potential interest for publication. We have changed our manuscript in line with the given considerations, and we address each comment individually below.

We hope to have further strengthened our manuscript and that it is now suitable for publication in PLOS ONE.

We look forward to hearing from you.

Kind regards,

Daniel Biermann, on behalf of all authors.

**Journal Requirements:**

**Comment 1:**

Please ensure that your manuscript meets PLOS ONE's style requirements, including those for file naming. The PLOS ONE style templates can be found at <https://journals.plos.org/plosone/s/file?id=wjVg/PLOSOne_formatting_sample_main_body.pdf> and <https://journals.plos.org/plosone/s/file?id=ba62/PLOSOne_formatting_sample_title_authors_affiliations.pdf>

**Answer 1**:

We revised our manuscript and edited Title page, Manuscript body and file names.

**Comment 2:**

Please provide additional details regarding participant consent. In the ethics statement in the Methods and online submission information, please ensure that you have specified (1) whether consent was informed and (2) what type you obtained (for instance, written or verbal, and if verbal, how it was documented and witnessed). If your study included minors, state whether you obtained consent from parents or guardians. If the need for consent was waived by the ethics committee, please include this information.

If you are reporting a retrospective study of medical records or archived samples, please ensure that you have discussed whether all data were fully anonymized before you accessed them and/or whether the IRB or ethics committee waived the requirement for informed consent. If patients provided informed written consent to have data from their medical records used in research, please include this information.

**Answer 2:**

We edited our paragraph on ethical approval. We write now: For the study, all authors obeyed the Declaration of Helsinki. The local ethics committee waived the requirement for informed consent since we are reporting a retrospective study of anonymized samples (ethics committee of the medical association Hamburg, Germany, approval number: WF-063/21). A copy of the certificate is uploaded to the EditorialManager.

**Comment 3**:

We note that you have indicated that data from this study are available upon request. PLOS only allows data to be available upon request if there are legal or ethical restrictions on sharing data publicly. For more information on unacceptable data access restrictions, please see <http://journals.plos.org/plosone/s/data-availability#loc-unacceptable-data-access-restrictions>.

In your revised cover letter, please address the following prompts:

a) If there are ethical or legal restrictions on sharing a de-identified data set, please explain them in detail (e.g., data contain potentially sensitive information, data are owned by a third-party organization, etc.) and who has imposed them (e.g., an ethics committee). Please also provide contact information for a data access committee, ethics committee, or other institutional body to which data requests may be sent.

b) If there are no restrictions, please upload the minimal anonymized data set necessary to replicate your study findings as either Supporting Information files or to a stable, public repository and provide us with the relevant URLs, DOIs, or accession numbers. For a list of acceptable repositories, please see <http://journals.plos.org/plosone/s/data-availability#loc-recommended-repositories>.

We will update your Data Availability statement on your behalf to reflect the information you provide.

**Answer 3:**

There are no restrictions for uploading our data. We included our data set in the Supporting Information section.

**Comment 4:**

Your ethics statement should only appear in the Methods section of your manuscript. If your ethics statement is written in any section besides the Methods, please delete it from any other section.

**Answer 4:**

We corrected this, and the ethics statement appears now in the Method section.

**Review Comments to the Author**

**Reviewer #1**

**Comment 1:**

In Introduction, line 53 please add "and" after " leave the operating room with an open chest" instead of comma; Also line 55 and 56 "mediastinitis" is technically a local SWI. I would change the "local SWI" to "superficial wound infection"

**Answer 1:**

Thank you for the advise, we corrected these sentences.

**Comment 2:**

Authors have compared groups "with and without SWI" and "Primary vs Delayed sternal closure" but I feel it would be important to compare the patients in delayed sternal closure group (n=163) who had SWI (n=24) and who did not (n= 139), to identify risk factors i.e. time to close, any chest opened at bed side for bleeding or tamponade, duration of antibiotics etc.

**Answer 2:**

Thank you for this suggestion. We compared those two groups you mentioned and added another table, as seen in the manuscript. The only significant risk factor in this analysis was a younger age at the time of surgery.

**Reviewer #2**

**Comment 1:**

With open chest, the authors used ampicillin/sulbactam till the sternum is closed. This seems to be not as a broad spectrum as other combinations that are commonly used such as “Cefepime/Vancomycin” especially against virulent microorganisms such as pseudomonas. Please clarify the reason for that choice among other antibiotic combinations.

**Answer 1:**

Thank you for your comment. In the absence of positive cultures and with no signs of infection, we prefer using a broad-spectrum antibiotic treatment with minimal risk for adverse effects like kidney function impairment. Nonetheless, we extend therapy according to guidelines once the microbiological smears have been evaluated. We discuss this comment in terms of lacking evidence in the Discussion section of the manuscript.

**Comment 2:**

The use of Gentamycin for chest irrigation at the time of closure is not evidence-based, especially the Gentamycin has a narrow spectrum of coverage. Did the author evaluate if this decreased risk of wound infection or not? Any evidence to support its use?

**Answer 2:**

We totally agree with you. Currently, the evidence regarding antibiotic agents, dosages, application protocols and SWI definitions vary widely throughout studies. However, our standard-of-care-practice for wound closure based on the findings from a RCT from Fridberg et al. The researcher showed that local collagen-gentamicin reduced the risk for postoperative sternal wound infections in adults. (Friberg O, Svedjeholm R, Söderquist B, Granfeldt H, Vikerfors T, Källman J. Local gentamicin reduces sternal wound infections after cardiac surgery: a randomized controlled trial. Ann Thorac Surg. 2005 Jan;79(1):153-61; discussion 161-2. doi: 10.1016/j.athoracsur.2004.06.043. PMID: 15620935.) In everyday practice, we no longer use gentamycin rinsing solution in children and use only warm saline solution for cleaning the situs.

**Comment 3:**

Those on Extracorporeal membrane oxygenator support (ECMO), was there any change in their antibiotic coverage? Did they remain on antibiotics during the entire support period?

**Answer 3:**

Antibiotic treatment (ampicillin/sulbactam) was given throughout the open-chest phase and 48 hours thereafter. In patients on ECMO during the entire therapy period. Antibiotics were adjusted as necessary after review of the swabs.

**Comment 4:**

Any utilization for vacuum-assisted wound closure for any of these patients?

**Answer 4:**

We did not use any vacuum-assisted devices in small children (<1 year). In larger children, we have had some good experiences with vacuum dressing in recent years. This experience will be published separately in the future.

**Comment 5:**

What practical measures the author will take or advise to further decrease risk of wound infection after pediatric cardiac surgery?

**Answer 5:**

Thank you for this good question. As described in the manuscript, we think that our study should not claim to make general recommendations. We have significantly deepened the topic of sterility in PCICU in recent years and now use sterile gloves and sterile gel during ultrasound examinations. In addition, we have become more aggressive in treating superficial wound infections and defects directly surgically and additionally with antibiotics. This has meant that we have not had any mediastinitis or deep wound healing problems in the age group described.

**Comment 6:**

Have the authors evaluated other wound-related complications such as: breakdown in the incision, wound drainage without actual infection, and fat necrosis? We believe these complications are even more common than actual wound infection especially in neonates and small infants.

**Answer 6:**

Thank you for the question. In our cohort, wound-related complications such as suture dehiscence and wound drainage were summarized as superficial wound infection, if there were additional signs of infection including redness, swelling, warmer skin and / or fever. Mostly, it was wound dehiscence in the lower or upper wound pole leading to surgical revision in our experience. Here (in the last years) a reduction of the frequency was achieved by using SteriStrips. This seems to provide additional safety especially when newcomers to the profession perform sternal or skin closures. We have not observed fatty tissue necrosis in the age group under one year. However, the author team also thinks that this etiology is the most common cause of SWI in older children and adults.

**Comment 7:**

The authors should look into the relationship between the wound infection risk and the duration of open chest.

**Answer 7:**

In our cohort, we were unable to show any correlation in this respect, which is probably due to statistical reasons. It could be because patients with very long open sternal wounds partly died before a wound-healing disorder became evident. However, we also think that this is an important aspect. We compared the periods of open chest in the group of patients with DCS and included it in the new table.
